# A Rare Case of Mandibular *Aspergillus* Osteomyelitis in an Immunocompetent Patient

**DOI:** 10.3390/dj10110213

**Published:** 2022-11-09

**Authors:** Isabel Schausltz Pereira Faustino, Joab Cabral Ramos, Bruno Augusto Linhares Almeida Mariz, Erofili Papadopoulou, Maria Georgaki, Nikolaos G. Nikitakis, Pablo Agustin Vargas, Alan Roger Santos-Silva, Marcio Ajudarte Lopes

**Affiliations:** 1 Department of Oral Diagnosis, Piracicaba Dental School, University of Campinas, Piracicaba 13414-903, Brazil; 2 Department of Oral Medicine & Pathology and Hospital Dentistry, School of Dentistry, National and Kapodistrian University of Athens, 11527 Athens, Greece

**Keywords:** aspergillosis, *Aspergillus*, osteomyelitis, mycoses, mandible

## Abstract

Aspergillosis is a fungal infection caused by *Aspergillus* species, which is contracted through spores that colonize the respiratory tract, causing rhinosinusitis and pulmonary infections. Oral aspergillosis is rare and, when present, may cause soft tissue and bone destruction, generally in immunodeficient patients. Mandibular *Aspergillus* osteomyelitis is even rarer, with few cases reported in the literature. A 57-year-old Caucasian woman was referred for the evaluation of painful recurrent swelling in the anterior mandibular alveolar ridge, with purulent drainage, previously treated with multiple surgical debridement procedures and antibiotics without success. The patient was otherwise systemically healthy. Surgical debridement was performed and histopathological examination showed osteomyelitis associated with *Aspergillus* species. Therapy with oral itraconazole (400 mg per day) was administered for 3 months, resulting in complete resolution. No recurrence was detected after 15 years of follow-up. The patient was rehabilitated with dental implants. In conclusion, non-bacterial microorganisms, such as *Aspergillus*, should be considered in cases of mandibular osteomyelitis that do not heal after surgical debridement and antibiotic therapy.

## 1. Introduction

*Aspergillus* species are a genus of fungi, ubiquitous throughout nature. They are a collection of around 200 soil-dwelling and airborne mold fungi, also found on decaying organic material. Some of these fungi are widely used beneficially for the fermentation of soybeans, rice, grains, and potatoes, as well as the large-scale production of enzymes, organic acids, and bioactive compounds [1]. Among all these diverse species, some fungi are pathogenic; *Arpergillus fumigatus* and *Aspergillus flavus* are the most common species capable of causing infections in humans and animals. Aspergillosis can cause a broad range of clinical diseases, ranging from asymptomatic infection and colonization to life-threatening invasive aspergillosis. Commonly, this fungal infection is established through spores that colonize the respiratory tract, causing rhinosinusitis and pulmonary infections [2,3]. 

Generally, the major risk factor to contract aspergillosis is any form of deficiency in the host immune system, including, but not limited to, hematological malignancies, bone marrow transplantation, aplastic anemia, acquired immunodeficiency syndrome, long-term steroid, use and, recently, COVID-19 infection [4]. *Aspergillus* hyphae can invade blood vessels, causing secondary thrombosis and, consequently, tissue necrosis [2]. Bone-invasive aspergillosis is a rare form of osteomyelitis and can cause significant morbidity and even death [3]. Oral aspergillosis is uncommon and, when present, may cause soft tissue and bone destruction [5,6,7,8,9]. 

Although aspergillosis is uncommon in immunocompetent patients, some clinical cases of oral aspergillosis in healthy hosts have been described and most of them are located in the maxillary sinus [6,7,10,11]. Therefore, this article aims to describe a rare and challenging case of mandibular *Aspergillus* osteomyelitis in an immunocompetent individual, highlighting the clinical manifestation, diagnosis, and management.

## 2. Case Report

A 57-year-old Caucasian woman presented with a 17-month history of persistent infection on the left side of her mandible that occurred after tooth extraction. The patient reported recurrent swelling, pain, and purulent drainage. She had a history of five surgical debridement procedures and the use of broad-spectrum antibiotics without resolution. Although the patient could not specify when the debridement procedures were performed, they took place over 17 months. Previous medical history was unremarkable and the patient denied medication use and any condition of immunosuppression, confirmed by laboratory exams. 

**Figure 1:**
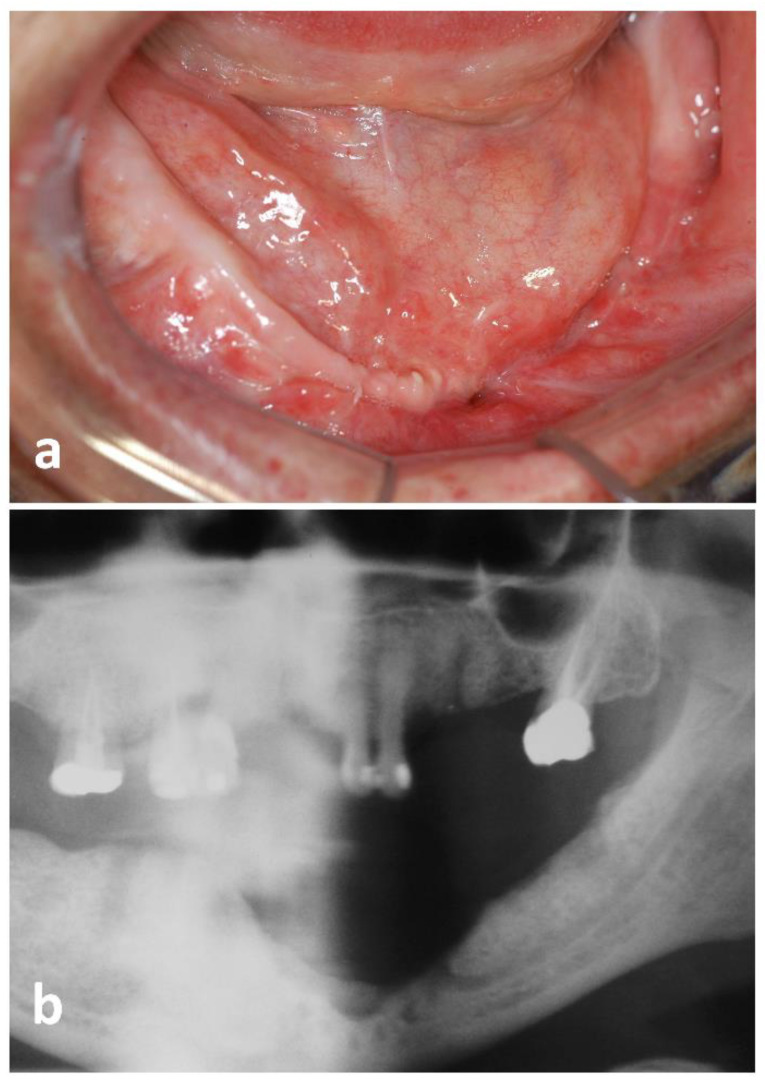
Clinical features of mandibular *Aspergillus* osteomyelitis. (**a**) Oral fistula and diffuse swelling on the lower left alveolar ridge. (**b**) Radiographic aspect, showing marginal bone resorption with poorly defined borders caused by *Aspergillus* infection in the mandible.

**Figure 2:**
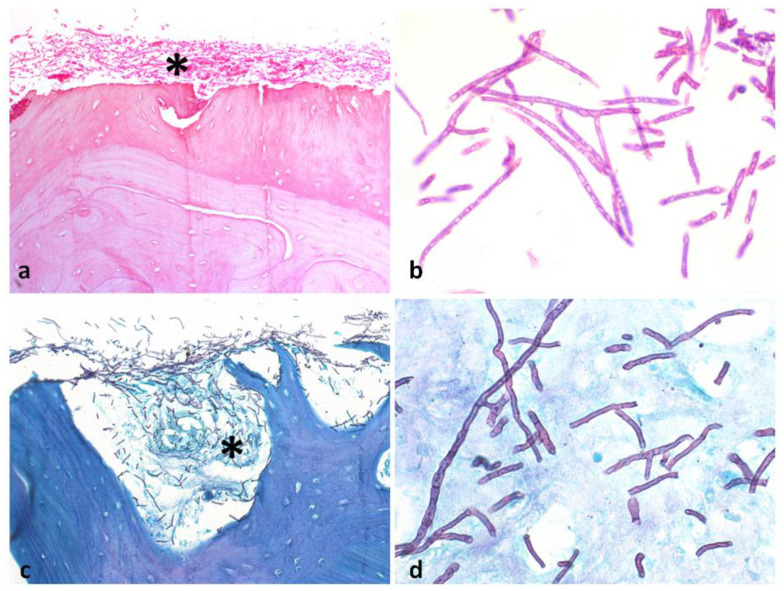
Histopathological features of mandibular *Aspergillus* osteomyelitis. (**a**) Necrotic bone fragment showing large number of fungal hyphae at the periphery (HE, orig. mag. 20×). (**b**) High-power photomicrograph shows septate hyphae with dichotomous 45° branching, characteristic of *Aspergillus* species (HE, orig. mag. 100×). (**c**,**d**) Grocott–Gomori methenamine silver stain highlighting *Aspergillus* hyphae colonizing the necrotic bone (orig. mag. 20× (**c**) and 100× (**d**)). Asterisks indicate septate *Aspergillus* hyphae.

Oral examination revealed a fistula and diffuse swelling on the lower left alveolar ridge (Figure 1a). Significant bone loss was also observed, caused by the previous surgical interventions. No cervical lymphadenopathy was noted. Panoramic radiograph showed marginal bone resorption with poorly defined borders on the left side of the mandible, which was in close contact with the mental foramen (Figure 1b). Based on the history of five surgical debridement procedures accompanied by broad-spectrum antibiotic therapy, a new surgical debridement was performed and a tissue sample was collected. The histopathological examination showed necrotic bone fragments and osteomyelitis associated with a large number of septate hyphae, with dichotomous acute angle branching, consistent with *Aspergillus* infection (Figure 2a,b). Fungal hyphae were positive for Groccott-Gomori methenamine silver stain (Figure 2c,d). Laboratory exams and chest radiograph were requested but did not reveal any pathologic findings. Although, after the surgical procedure, the patient had no clinical indication of infection, she was referred to an infectious disease specialist, who prescribed oral itraconazole (400 mg per day) for 3 months. A close follow-up was instituted and complete resolution was achieved 15 days after surgical manipulation to diagnostic confirmation.

**Figure 3:**
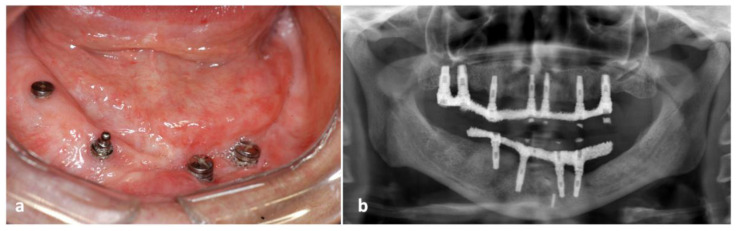
Clinical condition with 10 years of follow-up (**a**) and radiographic aspects after 15 years of follow-up (**b**). Clinical and radiographic features showing no evidence of recurrence or disease relapse.

Afterwards, the patient was subjected to a bone graft, dental implant placement, and rehabilitation with prosthesis. The patient remained in clinical and radiographic evaluation on a regular basis, without evidence of recurrence or disease relapse after 15 years of follow-up (Figure 3a,b). 

## 3. Discussion

The term aspergillosis refers to a spectrum of disease manifestations caused by fungus *Aspergillus* spp. These manifestations are dependent on factors related to the host and immune response and the site of the infection [4]. *Aspergillus* species are the most common fungi in the environment, and aspergillosis is the second most prevalent opportunistic mycosis [12]. *Aspergillus* is commonly found in soil and decaying vegetative material and secretes several proteolytic enzymes. The prevalence of *Aspergillus* species is influenced by regional characteristics in precipitation, humidity, and temperature. It is also believed that these regional particularities may also have a correlation with infection in humans [13]. However, generally, the main species involved in infections are *Aspergillus fumigates*, *Aspergillus flavus*, *Aspergillus niger*, and *Aspergillus terreus*. Infection by the fungus occurs through the inhalation of spores that reach the lungs and can develop locally or disseminate to adjacent or distant sites [4].

Generally, the main site of *Aspergillus* infection is the lung, which occurs due to spores’ ability to colonize the respiratory tract, and, in this context, other contiguous structures are also usually affected, such as pleura and lymph nodes [4,7,14]. Aspergillosis can manifest in two different ways: non-invasive and invasive. The non-invasive form is mainly characterized by aspergilloma, allergic bronchopulmonary aspergillosis, and the chronic form of pulmonary aspergillosis (considered a semi-invasive form). Invasive forms are mainly invasive pulmonary aspergillosis and tracheobronchial aspergillosis. It is still possible to identify extrapulmonary aspergillosis, such as ocular aspergillosis, osteomyelitis due to aspergillosis, and central nervous system aspergillosis [4]. In the head and neck region, the maxillary sinus is the most commonly affected site of infection by *Aspergillus* [15]. Maxillary sinus aspergillosis may occur as the primary site or manifest as a disseminated infection of pulmonary origin. An aspergilloma (a fungus ball) may develop within the sinus cavity, or invasive disease may be seen [4]. In some cases, the infection may progress to the oral cavity, mainly affecting adjacent structures, such as the hard and soft palate [6,7,16]. For the most part, *Aspergillus* as a pathogen cannot actively penetrate undamaged mucous membranes or intact skin, as it lacks keratolytic enzymes, and therefore deep structures are less affected [17]. In this context, oral aspergillosis may be caused by previous oral infections, endodontic treatment of upper teeth, or infected root canal cements or endodontic materials, favoring fungal growth [18]. *Aspergillus* osteomyelitis is a rare form of aspergillosis mainly affecting the ribs, vertebrae, and cranium, resulting from contiguous spread from adjacent infection, direct inoculation from surgery or trauma, or hematogenous spread [3]. Our case describes a peculiar case of jawbone aspergillosis, located in the mandible, which possibly emerged after dental extractions. It is also important to mention that the current patient was immunocompetent, making this case even rarer. 

Orofacial aspergillosis presents as a spectrum of chronic sinusitis, antral aspergilloma, allergic sinusitis, invasive antral aspergillosis, and oral lesions [12,19]. Generally, oral involvement can occur as part of disseminated lung infection, or occasionally as an extension of maxillary sinus infection, and these manifestations are mostly described in patients with some degree of immunosuppression [20]. Interestingly, we describe a case of oral aspergillosis limited to the mandible, without any systemic signs or symptoms, in an immunocompetent patient, but with a history of previous tooth extraction at the site of infection.

Classically, oral aspergillosis presents two clinicopathological stages [21]. The initial stage shows isolated violaceus areas in the gingiva, which might progress to grayish necrotic ulcers with a pseudomembrane. Generally, the ulcer’s base shows vascular invasion, which can lead to the late phase characterized by alveolar bone destruction [20]. Our case presented as painful mandibular swelling draining pus, with no area of necrosis, despite its protracted 17-month course. Purulent drainage and radiographic examination indicated bone involvement, which was confirmed microscopically as osteomyelitis.

Usually, osteomyelitis of the jaws is related to bacterial infections and typically presents as edema, fistula, and pain. Increased bone density and decreased collateral blood supply make the mandible the most affected site [22]. Fungal osteomyelitis is rare, caused most frequently by Candida infection [17]. *Aspergillus* osteomyelitis is even rarer, but has been mentioned in the English-language literature. A review of 310 cases of *Aspergillus* osteomyelitis demonstrated that only 18% were located in the base of the skull, paranasal sinuses, and jaw [23]. Few studies of mandibular aspergillosis have been published, mainly in immunocompromised patients [2,5,7,14,24] (Table 1). Mandibular *Aspergillus* osteomyelitis is related to gingival necrosis, swelling, and bone sequestration, and to systemic symptoms, such as fever or concomitant pulmonary aspergillosis [2,5,7,14,24]. To the best of our knowledge, this is the first case in which the clinical manifestation is limited to swelling and fistula, which might be explained by the patient’s intact immunologic status.

It is important to emphasize that the literature describes that some *Aspergillus* species grow especially when no other bacteria are present to suppress their growth [3]. The present case reports a patient who was initially treated as a bacterial alveolitis, receiving surgical debridement and antibiotic therapy. Over the five management attempts with antibiotic use, the balance between local bacteria and *Aspergillus* may have been impaired and the fungal infection ran its course without clinical resolution. This condition reinforces the importance of recognizing the microorganism involved in the infection for proper treatment. In addition, it is also relevant to always emphasize the need to send all material removed from the patient for microscopical analysis, including tissue collected through curettage in suspected osteomyelitis, a step that was not performed in the five previous surgical interventions to which the current patient was submitted.

Diagnostic confirmation can be made by microbiological and histopathological analysis of the material, using periodic acid Schiff (PAS) or Groccott–Gomori methenamine silver stains [12,25]. Some less invasive techniques can also be helpful in the diagnosis of aspergillosis, such as the investigation of circulating antigens and components of the fungal wall in the patient’s plasma. However, the results may not be specific [4,26]. Species identification is not always necessary, but, in cases resistant to standard antifungal therapy, recognition should be made to determine specific drug therapy [4]. If the diagnosis is confirmed, oral aspergillosis needs a multidisciplinary approach, and systemic involvement might be evaluated. Oral lesions are treated with systemic antifungals and local surgical debridement [20]. Since the current patient presented normal laboratory exams and chest radiograph, treatment consisted of surgical debridement and oral systemic itraconazole, which was prescribed by a physician specializing in infectious diseases. In the present case, the identification of the species was not necessary and the disease responded very well to the implemented therapy. The patient was eventually rehabilitated with dental implants and remains in continuous follow-up, without any evidence of recurrence 15 years after treatment.

Risk factors for aspergillosis are widely known [4]. Immunosuppression by bone marrow transplantation renders aspergillosis the main opportunistic fungal infection for this group of patients [27]. The number of cases of aspergillosis is increasing, and this may be due to the fact that the number of immunosuppressed people has increased due to new immunosuppressant and chemotherapeutic regimens. In addition, it is important to note that new risk factors may arise with the emergence of new diseases or therapies that directly impact the body’s immune function [4]. Patients admitted to intensive care have recently been indicated as a potential risk population for aspergillosis, mainly those suffering from chronic obstructive pulmonary disease under corticosteroid use, structural lung disease, and acute respiratory distress syndrome [28]. Importantly for the current global health situation, viral respiratory infections have also been associated as a risk factor for *Aspergillus* infection [29,30].

Recently, it was proposed that patients infected with SARS-CoV-2 should be considered likely to develop invasive aspergillosis, known as COVID-19-associated pulmonary aspergillosis [29]. It is believed that damage to the epithelium, immune dysregulation, and impaired ciliary clearance can lead to tissue invasion by *Aspergillus*. Added to this, these patients may receive dexamethasone, baricitinib, and tocilizumab, which are immunomodulatory medications [4]. The current case describes a mandibular osteomyelitis diagnosed 15 years ago and, interestingly, for a period of 16 years, to the best of our knowledge, there were no reports of mandibular *Aspergillus* osteomyelitis. Nonetheless, recently, some cases of osteomyelitis by aspergillosis in the facial bones have been described in the literature in patients who were affected by COVID-19 [31]. Specifically in the mandible, one paper described two cases of osteomyelitis, but only in one patient the presence of *Aspergillus* was specified as the cause of bone infection [24]. Thus, due to the new consequences related to COVID-19, attention should also be paid to patients who have been under an intense regimen of corticosteroids and presenting infection-like conditions in the facial bones and/or soft tissue.

Ultimately, mandibular *Aspergillus* osteomyelitis may present with signs that can be misdiagnosed as bacterial osteomyelitis or alveolitis. Thus, other microorganisms, such as *Aspergillus*, should be considered in cases of bone infections after dental extractions that do not heal after surgical debridement and antibiotic therapy, even if the patient is immunocompetent [24]. Therefore, it is important to emphasize that, in cases of suspected alveolitis resistant to standard surgical and pharmacological therapies, the microorganism involved must be investigated by culture or microscopic analysis.

## 4. Conclusions

Here, we present the case of an immunocompetent woman who, after a tooth extraction, developed a condition similar to alveolitis. Thus, only after five unsuccessful surgical and drug interventions using antibiotics, the patient was referred to our service specializing in oral diagnosis. In the five previous interventions, surgical specimens were not collected for analysis and, therefore, we performed a new surgical debridement, which was sent for histopathological analysis and showed the presence of *Aspergillus* spp. in the necrotic bone. Based on the histopathological diagnosis, the patient received the appropriate treatment with antifungal medication and systemic evaluation.

In general, in cases of antibiotic-resistant alveolitis or osteomyelitis, aspergillosis must be suspected and the diagnostic criteria must be carefully followed for the correct treatment, keeping in mind that some specialists may have extensive experience in dealing with such cases. Therefore, we believe that this report has the potential to facilitate the diagnosis of aspergillosis in gnathic bones that otherwise may have remained undiagnosed.

## Figures and Tables

**Table 1:** Review of mandibular *Aspergillus* osteomyelitis reported in the English-language literature.

Author	Year	Sex	Age	Risk Factors	Oral Manifestation	Systemic Manifestation
Hovi et al. [5]	1996	F	5	Leukemia	Gingival necrosis and swelling	Fever
Filippi et al. [2]	1997	M	36	Leukemia	Gingival necrosis, bone sequestration	Pulmonary aspergillosis
Sandhu and Kaur [7]	2003	F	35	None	Gingival necrosis	Pulmonary aspergillosis
Lador et al. [14]	2006	F	42	Leukemia	Gingival and alveolar mucosa necrosis	Fever
Chugh et al. [24]	2022	M	64	Post-COVID-19	Alveolar mucosa and bone necrosis	None
Current	2022	F	57	None	Swelling and fistula	None

## Data Availability

For data information, contact the corresponding author.
